# On polyhedral graphs and their complements

**DOI:** 10.1007/s00010-022-00902-5

**Published:** 2022-08-18

**Authors:** Riccardo W. Maffucci

**Affiliations:** EPFL, MA SB Batiment 8, Lausanne, Switzerland

**Keywords:** Planar graphs, 3-connectivity, Polyhedra, Complements, Dual graph, Classification, 05C10, 51M20, 05C75, 05C30

## Abstract

We find all polyhedral graphs such that their complements are still polyhedral. These turn out to be all self-complementary.

## Introduction

### The problem

The problem we investigate combines two main ideas. Polyhedral graphs, or simply polyhedra, are 3-connected, planar graphs. This class of graphs is closely related to 3-dimensional topology and geometry, and the name comes from the fact that they are 1-skeletons of polyhedral solids in the sense of geometry (Rademacher-Steinitz’s Theorem, see e.g. [11, Theorem 11.6]). In what follows, we assume polyhedral solids to be convex, and consider them up to homeomorphism, i.e., up to their 1-skeletons being isomorphic graphs. It may be shown using only graph theory that there are only five regular polyhedral solids, namely the Platonic ones [12, Theorem 1.38].

Polyhedral graphs have several nice properties. They are the planar graphs that can be embedded in a sphere in a unique way (an observation due to Whitney, see e.g. [11, Theorem 11.5]). Specifically, the dual graph is always unique, and duals of polyhedra (in the sense of both graph theory and geometry) are also polyhedra (e.g., [11, Chapter 11]). We also record that all their regions (or ‘faces’) are delimited by cycles (elementary closed walks) [7, Proposition 4.26].

The other idea comes from a classical problem in graph theory, set by Harary: to find all graphs *G* such that a certain property is verified by both *G* and its complement graph $\overline{G}$ [5, Introduction]. Our problem is the following.

#### Question 1

Which pairs of complementary graphs $G,\overline{G}$ are both polyhedral?

#### Theorem 2

**Figure 1:**
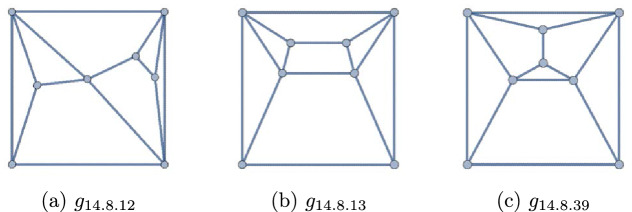
The only three solutions to Question 1

There exist exactly three polyhedral graphs such that their complements are polyhedral. These are all self-complementary. They are depicted in Fig. 1,^1^.

All solutions to Question 1 are (8, 14) graphs, of degree sequence

$$\begin{aligned} 4,4,4,4,3,3,3,3. \end{aligned}$$

This case promises to be the most interesting, due to the following.

#### Remark 3

If the polyhedron *G*, its dual, and its complement graphs are all of the same order and size, then *G* is an (8, 14) graph. To see this, we impose the following three conditions. If *G* and $G'$ have the same order, then the number of regions and vertices of *G* coincide. If $G,\overline{G}$ have the same size, then $$q=\frac{1}{2}\left( {\begin{array}{c}p\\ 2\end{array}}\right) $$. The third condition is Euler’s formula for planar graphs. Solving the resulting system, we get $p=8$ and $q=14$.

*Related problems* Planar graphs with planar complements were investigated in [3, 5]. In [13, Figure 3.1], there are the only three non-trivial, self-complementary, self-dual graphs. In this figure, graph A is $g_{14.8.13}$ of Fig. 1, C is $g_{14.8.12}$, while B is not 3-connected. By the way, $g_{14.8.39}$ in Fig. 1 is the only self-complementary, non-self-dual polyhedron, as we shall see in Sect. 3. In another related work, Ando-Kaneko [2] investigated the connectivity of complements of 3-connected graphs.

As mentioned, the Rademacher-Steinitz Theorem characterises all polyhedra in graph-theoretic terms. Certain subclasses of polyhedra have been similarly characterised only by their graph-theoretic properties [9]. For other works on 3-connected planar graphs and related topics see e.g. [1, 6, 14].

For connectivity properties shared by a graph and its complement, see e.g. [15, 16].

### Notation and conventions

We say that *G* is a (*p*, *q*) graph when *G* is a graph of order (number of vertices/points) *p* and size (number of edges/lines) *q*. The vertex and edge sets of *G* are *V*(*G*) and *E*(*G*) respectively. Vertices are denoted by $v_1,v_2,\dots ,v_p$. Their respective degrees are non-negative integers $d_1,d_2,\dots ,d_p$. The degree sequence of a graph is indicated by $d_1,d_2,\dots ,d_p$, or simply by $d_1d_2\dots d_p$ if there is no possibility of confusion.

A graph of order at least 4 is 3-connected if removing any set of 0, 1, or 2 vertices produces a connected graph. The complement of *G* is denoted by $\overline{G}$, and the dual by $G'$. The letters $\overline{p},\overline{q},p',q'$ indicate their orders and sizes accordingly. The number of regions of a planar graph is *r*, and of its complement and dual (if these are also planar) $\overline{r},r'$. The faces of a polyhedral graph are its regions. The faces are triangular, quadrilateral, pentagonal, ... if they are bounded by a cycle of length 3, 4, 5, ... respectively.

**Table 1:** Possible degree sequences for solutions of Question 1

Deg. sequence of G	Size q	Faces r	Deg. sequence of $\overline{G}$	Size $\overline{q}$	Faces $\overline{r}$
33333333	12	6	44444444	16	10
44333333	13	7	44444433	15	9
44443333	14	8	44443333	14	8

**Figure 2:**
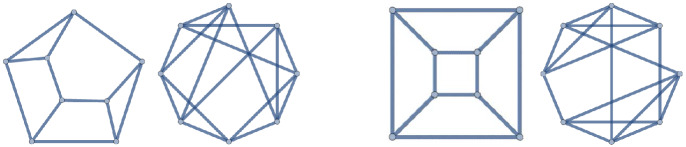
The two (8, 12) polyhedral graphs, and their complements

**Figure 3:**
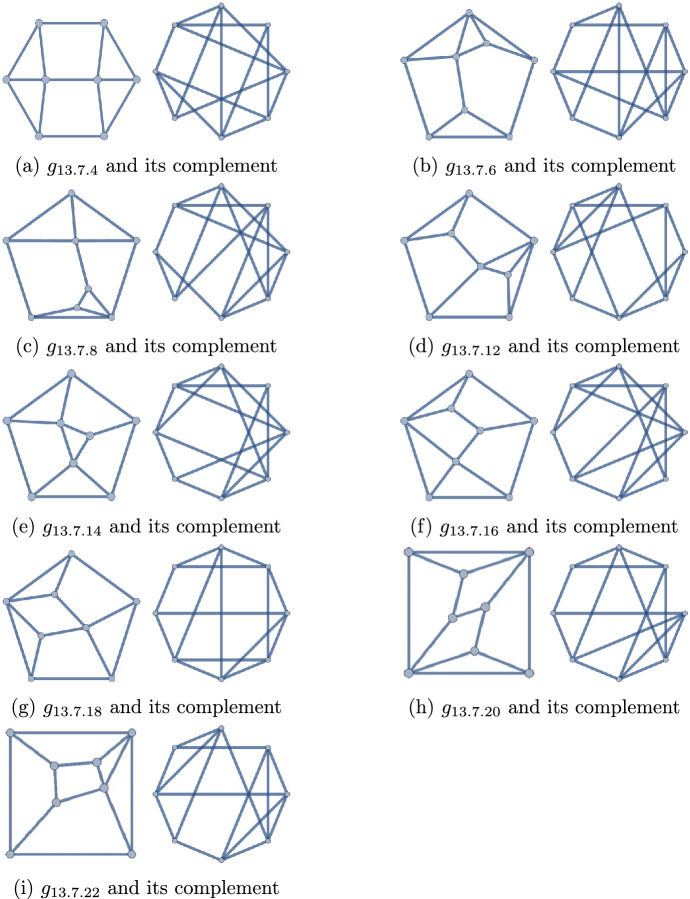
The nine polyhedra of degree sequence 44333333, and their complement graphs

**Figure 4:**
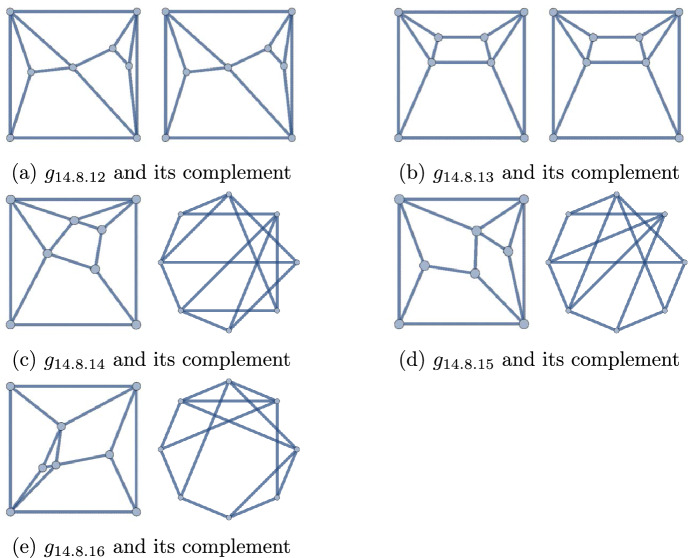
The self-dual polyhedra of degree sequence 44443333, and their complement graphs

**Figure 5:**
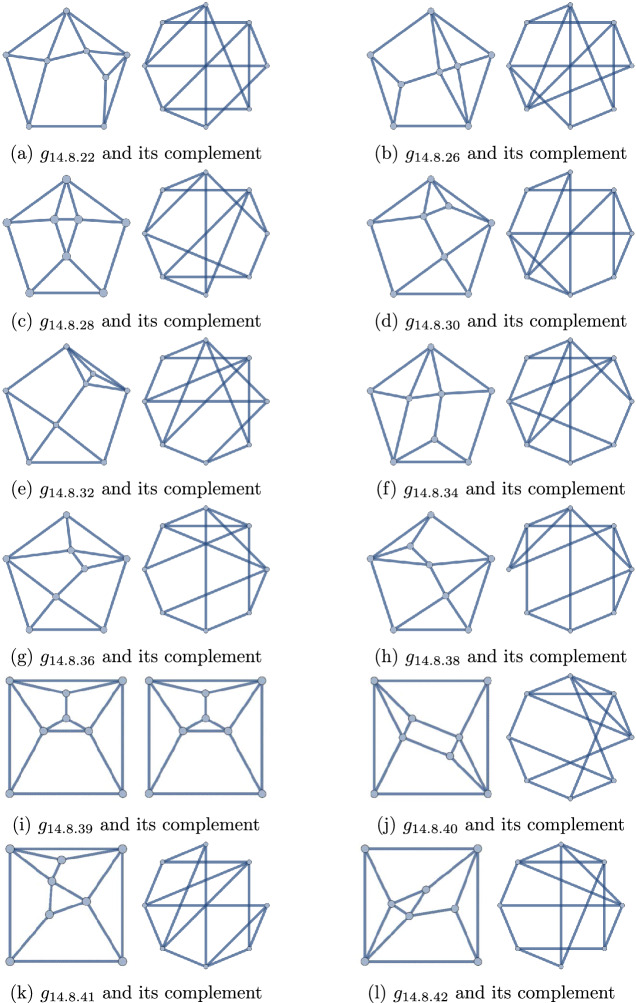
The non-self-dual polyhedra of degree sequence 44443333, and their complement graphs

## Setup

It is well-known that if *G* is a planar graph on at least 9 vertices, then $\overline{G}$ is non-planar [3]. It follows right away that Question 1 has a finite number of solutions.

On the other hand, we will now show that if $G,\overline{G}$ are polyhedra, then $p\ge 8$. Denote by

$$\begin{aligned} d_1,d_2,\dots ,d_p \end{aligned}$$

the (weakly decreasing) degree sequence of *G*. Since the graph is 3-connected, in particular one has

2.1 $$\begin{aligned} d_p\ge 3. \end{aligned}$$

Accordingly, the degree sequence of $\overline{G}$ is

$$\begin{aligned} p-1-d_p,p-1-d_{p-1},\dots ,p-1-d_1, \end{aligned}$$

with $$p-1-d_1\ge 3$$, i.e.

2.2 $$\begin{aligned} d_1\le p-4. \end{aligned}$$

By (2.1) and (2.2), $p\ge 7$. If $p=7$, then *G* is 3-regular, which is impossible due to the handshaking lemma. Therefore, $p\ge 8$, so that ultimately $p=8$.

Further, the above yields $d_1=4$ and $d_8=3$. The handshaking lemma now reduces our cases to those of Table 1, assuming w.l.o.g. that $q\le \overline{q}$. It will thus suffice to inspect the cases $q=12,13,14$. This will be done in the next section.

## Completing the proof

A theorem of Tutte [18, Theorem 6.1] states that if *G* is a polyhedron of size *q* that is not a pyramid, then either *G* or its dual $G'$ may be obtained from a polyhedron of size $q-1$ by connecting with an edge two non-adjacent vertices on the same face. This gives an algorithm to construct all polyhedra of size *q* from those of size $q-1$ (once we take duals, include pyramids, and check for isomorphic graphs). The polyhedra up to 8 faces were thus tabulated in [4] and [10]. In “Appendix 3”, we collect those up to 14 edges, for quick reference. Tables for the number of polyhedra for fixed small orders and sizes may also be found in [8]. See “Appendix 3” for the explanation of the convention used in this paper for notations such as 14.8.12.

There are only two (8, 12) polyhedra (see Fig. 9). For both, the complement is non-planar—see Fig. 2.

**Figure 6:**
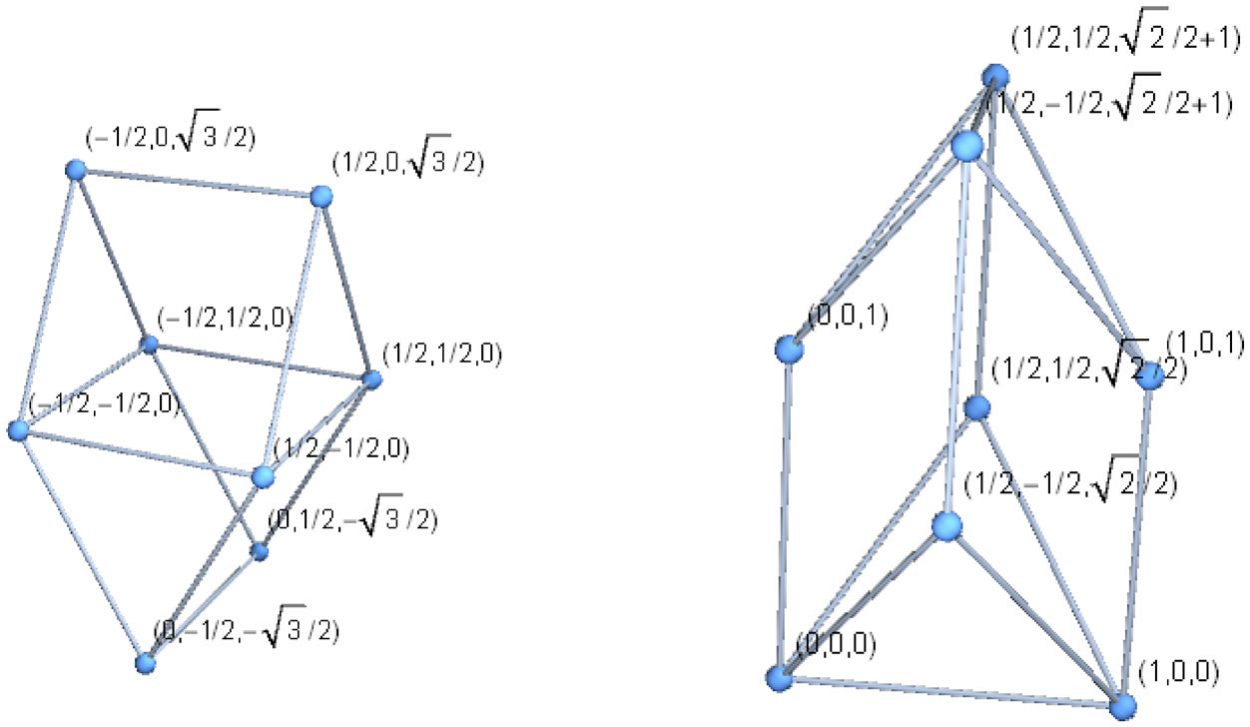
Embeddings of $g_{14.8.39}$ and its dual in 3-dimensional space, with all edges of unit length

There are eleven (8, 13) polyhedra, and nine of them have sequence 44333333 (Fig. 10). In Fig. 3, these are sketched together with their complement graphs. All of the complements are non-planar, hence we discard this case.

The forty-two (8, 14) polyhedra may be found in Figs. 12 and 13. Exactly seventeen of them have degree sequence 44443333. These are collected in Figs. 4 (self-duals) and 5 (non-self-duals). We find three solutions to Question 1, namely graphs $g_{14.8.12}$, $g_{14.8.13}$, and $g_{14.8.39}$.

As these lists of polyhedra are all exhaustive [4, 8, 10, 18], these three are the only solutions to Question 1. The proof of Theorem 2 is now complete. We note that $g_{14.8.39}$ is the only self-complementary, non-self-dual polyhedron.

As a remark of a different flavour, $g_{14.8.39}$ and its dual may be embedded in a 3-dimensional space so that all 14 edges have unit length, as in Fig. 6. For recent work on equilateral convex polyhedra, refer e.g. to [17].

## A: Tables of polyhedra

We choose the following ordering. Firstly, the tables are according to increasing size, rather than order. This is due to two main, related reasons. Understanding (*p*, *q*) polyhedra of $r=2+q-p$ faces (Euler’s formula), $p>r$, is no harder than studying the (*r*, *q*) of *p* faces, and then passing to the duals. In this sense, the complexity grows with *q* rather than *p*. Moreover, in this way each table lists dual pairs of polyhedra together (as they have the same size, but not necessarily the same order).

Self-duals are listed before dual pairs, and dual pairs are listed consecutively. Next, we sort by increasing number of vertices $\min \{|V(G)|, |V(G')|\}$. We have the inequalities

$$\begin{aligned} \frac{q+6}{3}\le p\le \frac{2q}{3} \end{aligned}$$

due to planarity ($q\le 3p-6$), and 3-connectivity (implying $\delta (G)\ge 3$, hence $q\ge 3p/2$ via the handshaking lemma).

All the above being equal, we sort by decreasing highest degrees of vertices, and then by decreasing highest degrees of vertices of the dual. If all the said criteria are equal, we list arbitrarily.

The graph name *x*.*y*.*z* for *G* is chosen accordingly, so that

$$\begin{aligned} |E(G)|=x \quad \text { and }\quad \min \{|V(G)|, |V(G')|\}=y. \end{aligned}$$

For fixed *x*, *y*, the polyhedra are distinguished by the value of *z*, starting from 1 up to the number of polyhedra satisfying $|E(G)|=x$ and $\min \{|V(G)|, |V(G')|\}=y$. As mentioned, our convention is that self-duals come before dual pairs, dual pairs are listed consecutively, and after that we sort by decreasing highest degrees of vertices, then by decreasing highest degrees of vertices of the dual, and all the above being equal, we list arbitrarily. For example, the 16 polyhedra (8 dual pairs) with $q=14$ and $p=7$ or 9 are listed as $14.7.1-14.7.16$ (Fig. 11), the dual of 14.7.1 being 14.7.2, and so forth.

### A.1: Size $q\le 12$

**Figure 7:**
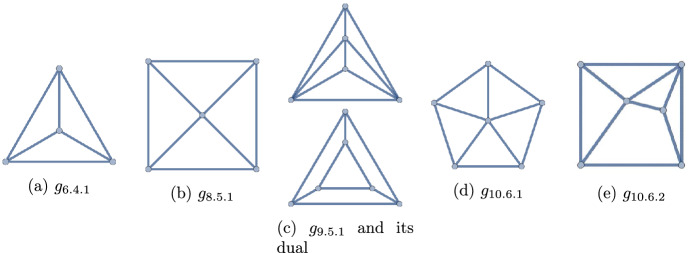
The 6 polyhedra with $q\le 10$

**Figure 8:**
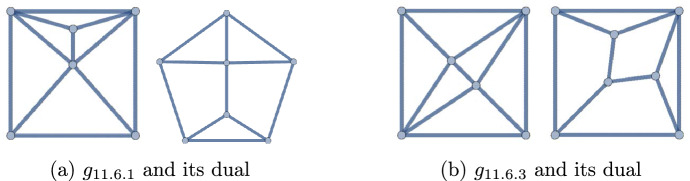
The 4 polyhedra with $q=11$

**Figure 9:**
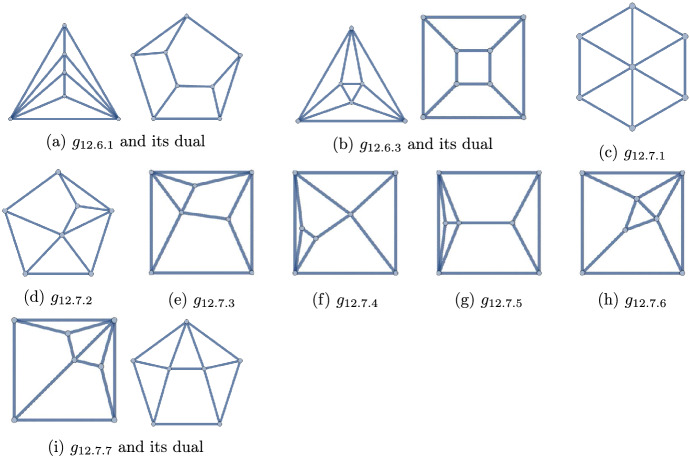
The 12 polyhedra with $q=12$

### A.2: Size $q=13$

**Figure 10:**
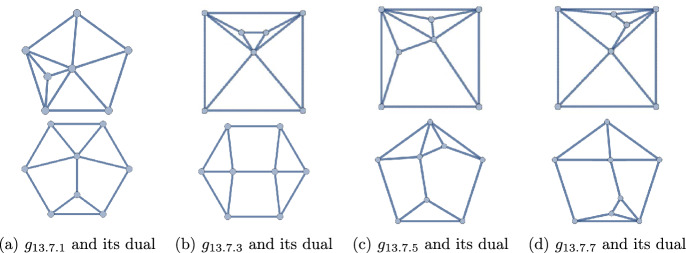

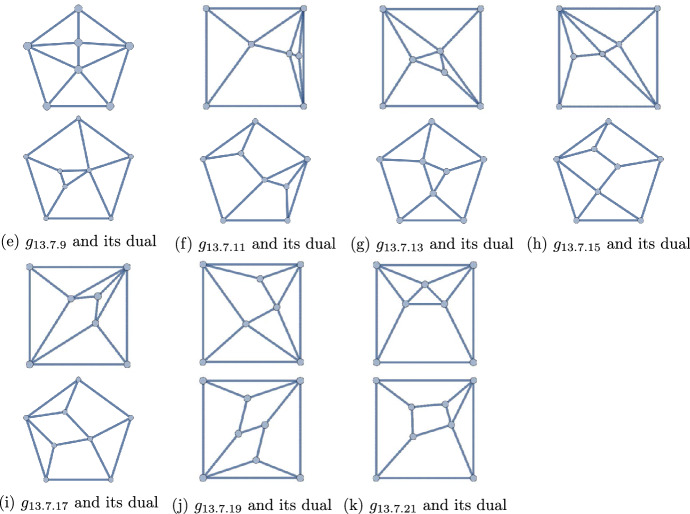
The 22 polyhedra with $q=13$

**Figure 11:**
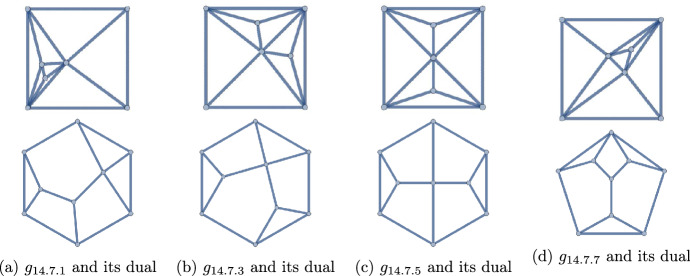

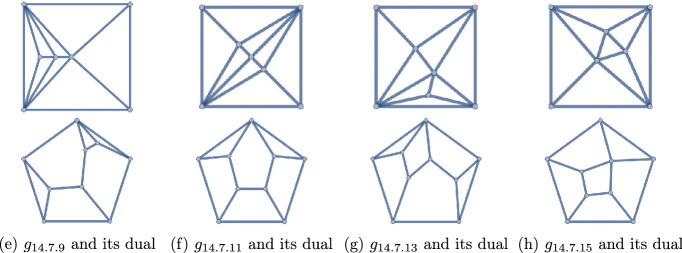
The 16 polyhedra with $q=14$ and $p=7,9$

### A.3: Size $q=14$

**Figure 12:**
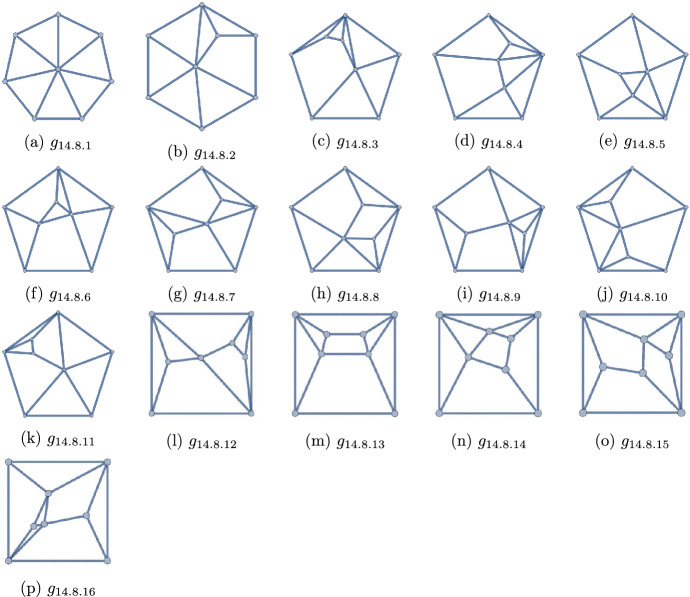
The 16 self-dual polyhedra with $q=14$ and $p=8$

**Figure 13:**
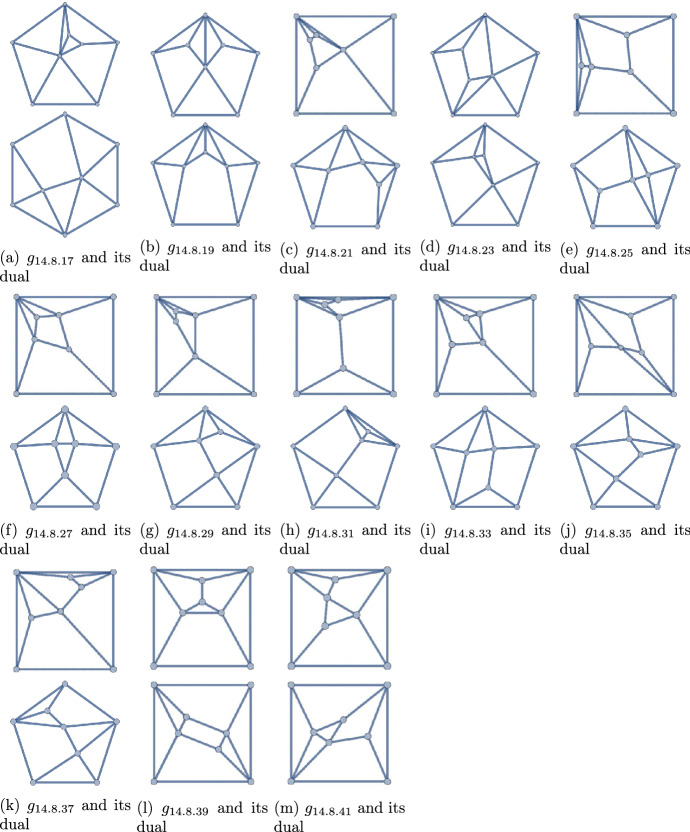
The 26 non-self-dual polyhedra with $q=14$ and $p=8$

## Data Availability

All data generated or analysed during this study are included in this article.

## Footnotes

1 See “Appendix 3” for the explanation of the convention used here for notations such as 14.8.12.
